# Numerical investigation on the role of check dams with bottom outlets in debris flow mobility by 2D SPH

**DOI:** 10.1038/s41598-022-24962-4

**Published:** 2022-11-28

**Authors:** Hao Shi, Yu Huang, Dianlei Feng

**Affiliations:** 1 Department of Geotechnical Engineering, College of Civil Engineering, Tongji University, Shanghai, 200092 China; 2 Key Laboratory of Geotechnical and Underground Engineering of the Ministry of Education, Tongji University, Shanghai, 200092 China; 3 Department of Hydraulic Engineering, College of Civil Engineering, Tongji University, Shanghai, 200092 China

**Keywords:** Natural hazards, Engineering

## Abstract

Check dams with bottom outlets are widely used in debris flow gullies to minimize the damage caused by debris flows. However, the bottom size is often based on empirical criteria due to the lack of knowledge of the interaction between the debris flow and the check dam with the bottom outlet. In this study, the interaction between a viscous debris flow and check dams with bottom outlets is investigated via flume tests using 2D smoothed particle hydrodynamics. The normalized height of the bottom outlet is varied from 0 to 1, and slope angles from 15 to 35° are considered. Based on the numerical results, the jump height decays with the increasing normalized height of the bottom outlet and this trend can be approximated by a power law function. When the normalized height of the bottom outlet is less than 0.15, the performance is similar to that of a closed check dam. The flow regulation and sediment trapping functions of the check dam may fail when the normalized height of the bottom outlet is greater than 0.6. These results show that the energy breaking, flow regulation, and sediment trapping functions of check dams with bottom outlets operate well when the normalized height of the bottom outlet is in the range 0.15–0.6. Even if model limitations require further efforts to validate the findings of this study, they provide a basis for the rational design of check dams with bottom outlets.

## Introduction

Human activities and natural disasters such as wildfires, earthquakes, and landslides lead to large numbers of dead, felled, or logged trees being scattered across the formation regions of debris flows. When a debris flow occurs, these trees are carried downstream^1^. The high velocity and huge volume of these flows often results in considerable damage to the lower reaches of a river basin. In addition, the costs associated with debris flows containing such driftwood include the loss of high-value wood and the maintenance of infrastructure in upstream regions^2^.

Various engineering countermeasures have been proposed to mitigate the damage caused by debris flows. Among them, check dams with openings have gradually increased in number as a means of improving the management of large woody debris flows^3^. Once a check dam has been installed in a debris flow gully, all the moving driftwood and sediment becomes trapped behind the dam before reaching more important structures downstream. However, the retention volume of a check dam may become fully filled after several debris flow events^4^. To enhance the sustainability of check dams, a bottom outlet is often positioned between the check dam and the debris flow channel bed^5^.

The main functions of check dams with bottom outlets are discharge regulation, sediment or driftwood trapping, and kinetic energy dissipation. These functions are provided by two mechanisms, namely the mechanical and hydraulic control of moving driftwood and sediment^6^. Mechanical control is often related to the jamming of openings when the characteristic scale of the driftwood and sediment exceeds the size of those openings^5^. Hydraulic control is intended to decrease the transport capability caused by water running back from the check dam^6^. Therefore, the size of the opening plays an important role in ensuring the functionality of the check dams.

Several experimental and numerical studies have been conducted to provide a scientific basis for the design guidelines of check dams with bottom outlets. Piton and Recking^3^ found that driftwood is likely to become trapped when the length of individual logs is twice the opening width, and showed that the trapping efficiency is negatively correlated with water discharge, the Froude number, and the outlet size. Schwindt^6^ found that the jamming probability is relatively high when the height of the outlet is less than the characteristic dimensions of the transported objects. Choi et al.^7^ conducted a series of flume tests to model the interaction between dry granular flows and check dams with bottom outlets. Based on this study, Shen et al.^8^ modeled the flume tests using discrete element method, and found that the Froude number and the normalized outlet size (the ratio between the outlet height ${H}_{c}$ and the particle diameter *D*) are two key considerations in assessing the jump height, impact force, energy-breaking efficiency, retention efficiency, and outflow rate. For dry monodisperse granular flows, clogging can be induced by the check dam if the height of the bottom outlet is 1.5 times the particle diameter. The retention efficiency and energy-breaking efficiency decrease as power functions of the increasing outlet size. To improve the performance of multiple-barrier systems, Ng et al.^9^ conducted a series of flume tests investigating the influence of the bottom outlet size of the first barrier on the overflow volumes and impact force in dual rigid barriers.

While these advances provide many useful suggestions for the design of check dams with bottom outlets, these studies are focused on the clogging of woody debris and dry granular flows. The interaction between viscous debris flows and check dams with bottom outlets has seldom been studied. In viscous debris flows, the stress is dominated by viscoplastic stress^10^. Therefore, a more comprehensive understanding of the interaction between viscous debris flows and check dams with bottom outlets is needed to provide a solid scientific basis for the design guidelines.

Rapid developments in numerical simulation methods have enabled quantitative studies of the dynamic interactions between viscous debris flows and rigid barriers. The main numerical simulation methods can be divided into two groups: grid-based methods and particle-based methods. Grid-based methods, such as the finite element method and finite difference method, are widely used in engineering. They may encounter grid distortion in the case of large-deformation problems^11^. Particle-based methods, such as smoothed particle hydrodynamics (SPH), discretize the continuum into a group of particles, thus avoiding the grid distortion caused by large deformations. Therefore, SPH is increasingly popular for studying debris flow–structure interactions. Dai et al.^12^ proposed a fluid–structure coupled numerical model to assess the impact force on rigid barriers. Li et al.^11^ studied the influence of the baffle shape on the debris flow impact force in a step-pool channel using SPH, and Yang et al.^13^ established a parallelized SPH model to study the impeding mechanism of baffles. Manenti et al.^14^ conducted a thorough study on interaction between a fast shallow landslide and downstream vertical rigid wall by SPH. The results show that SPH is a reliable tool for studying the dynamical interaction between debris flows and structures. Therefore, the in house code GeoSPH is adopted in this study to investigate the influence of the bottom outlet on viscous debris flow mobility.

The remainder of this paper is organized as follows: the problem is introduced and the governing equations are explained in “Physical problem and mathematical modeling” section. Then the δ-Plus-SPH scheme is described and verified in detail. All the numerical results are analyzed and discussed in the third section. Finally, some conclusions that could be useful for the design of check dams with bottom outlets are provided in “Conclusions” section.

## Physical problem and mathematical modeling

### Numerical flume model setup

**Figure 1:**
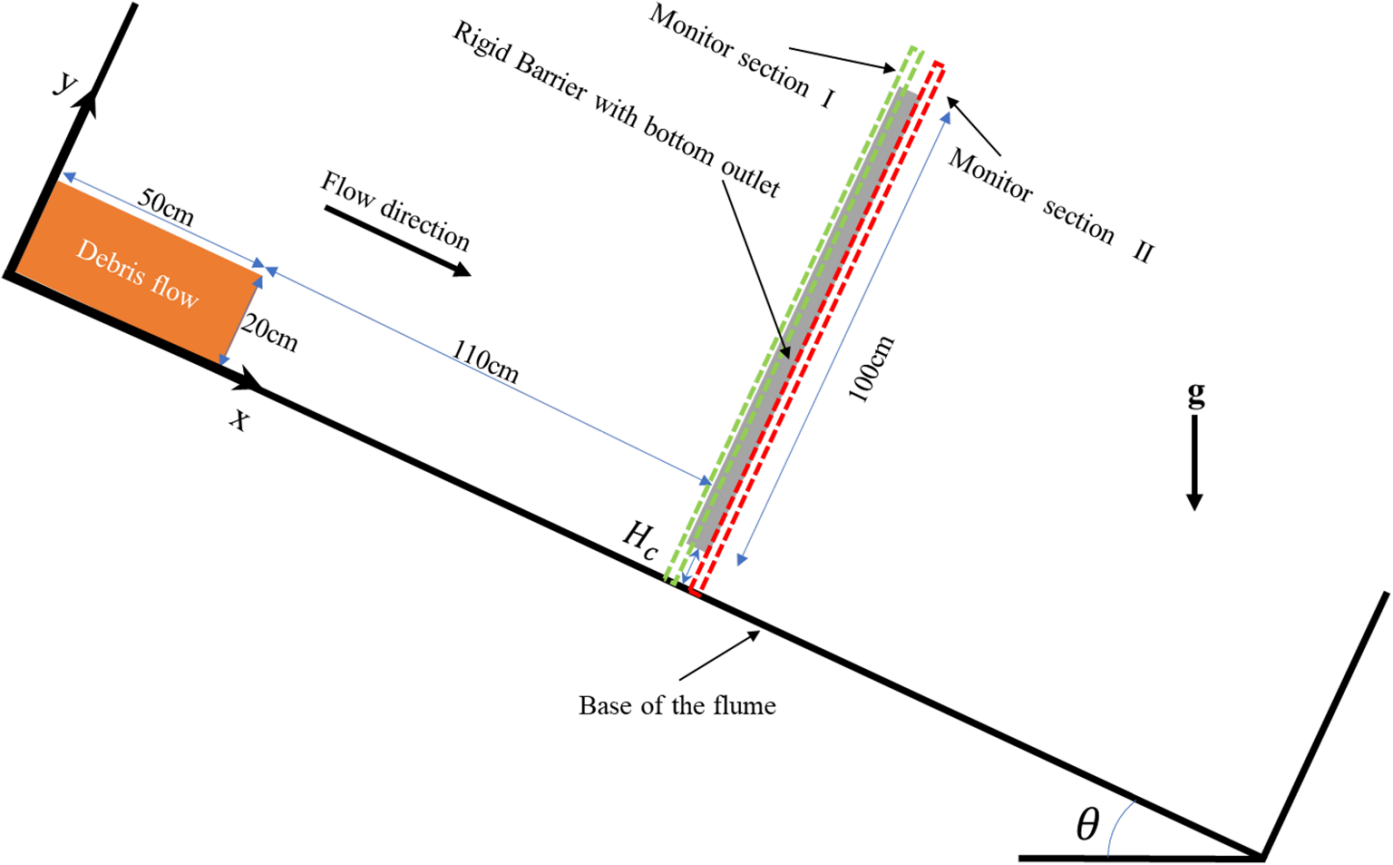
Numerical flume model setup.

Choi et al.^7^ conducted a series of flume tests to study the interaction between dry granular flow and check dam with bottom outlets. Then, based on their study, Shen et al.^8^ study the influence of Froude number and normalized outlet size on the performance of the check dams with bottom outlets by DEM. These two studies offer useful design guidelines for check dams with bottom outlets. The attention of these studies is paid to the mechanical control mechanism, while the hydraulic control mechanism is neglected. To investigate the hydraulic control mechanism of check dam with bottom outlets, we conduct a series of 2D numerical flume tests by SPH. The numerical setup of the flume test is a modified form of the experiment conducted by Choi et al.^7^. The basic configuration of the numerical flume model is shown in Fig. 1.

To study the hydraulic control mechanism of check dam with bottom outlets, a viscous debris flow (cross-section of 50 cm × 20 cm) is initially placed on a slope. A rigid barrier with a height of 100 cm and thickness of 5 cm is mounted 110 cm from the front of the initial position of the viscous debris flow. The height of the bottom outlet is defined as ${H}_{c}$. Various slope angles (*θ*) can be achieved by changing the angle between the body force and the y-axis.

The physical and rheological parameters of a natural debris flow that occurred in southern Italy are adopted in this paper^15^. The debris flow material is a fine-grained pyroclastic soil that was generated by the volcanic activity of Mount Somma-Vesuvius. The unit weight of the debris flow is 11.35 kNm^−3^.

The Herschel–Bulkley model has been widely used to describe the flow behavior of viscous debris flows^16–18^. In the Herschel–Bulkley model, the norm of deviatoric viscous stress tensor ${\varvec{\tau}}$ can be expresses as:

1 $$\begin{array}{*{20}c} {\left\{ {\begin{array}{*{20}l} {\left| {\varvec{\tau}} \right| = \tau_{y} + K\dot{\gamma }^{N}\quad if \dot{\gamma } \ne 0,} \hfill \\ {\left| {\varvec{\tau}} \right| \le \tau_{y} \quad otherwise,} \hfill \\ \end{array} } \right.} \\ \end{array}$$

where ${\tau }_{y}$ is the yield stress, $K$ is the consistency index, $N$ is the power law exponent, and $\dot{\gamma }={(2{\varvec{S}}:{\varvec{S}})}^{1/2}$ is the second invariant of the rate-of-strain tensor. Here, ${\varvec{S}}$ is the rate-of-strain tensor, which can be expressed as:

2 $$\begin{array}{*{20}c} {S = \frac{1}{2}\left( {\nabla {\varvec{u}} + (\nabla {\varvec{u}})^{T} } \right).} \\ \end{array}$$

To facilitate the numerical simulations, the apparent viscosity ${\eta }_{app}$ is introduced in the paper and the stress tensor ${\varvec{\tau}}$ can be expressed as:

3 $$\begin{array}{*{20}c} {\tau = 2\eta_{app} S,} \\ \end{array}$$

4 $$\begin{array}{*{20}c} {\eta_{app} = \frac{{\tau_{y} }}{{\dot{\gamma }}} + K\dot{\gamma }^{N - 1} ,} \\ \end{array}$$

where the shear rate can be expressed as $\dot{\gamma }={(2{\varvec{S}}:{\varvec{S}})}^{1/2}$.

Based on the experiments conducted by Schippa^15^, the Herschel–Bulkley model can describe the rheological behavior of this material with ${\tau }_{y}$, $K$, and $N$ set to 90 Pa, 4.526 Pa s^*N*^, and 0.795, respectively^15^.

The dynamics of the viscous debris flow can be expressed as:

5 $$\begin{array}{*{20}c} {\left\{ {\begin{array}{*{20}l} {\frac{D\rho }{{Dt}} = - \rho div\left( {\varvec{u}} \right),} \hfill \\ {\rho \frac{{D{\varvec{u}}}}{Dt} = - \nabla P + \nabla \cdot \tau + \rho g,} \hfill \\ \end{array} } \right.} \\ \end{array}$$

where $D()/Dt$ denotes the material derivative, $\rho$ and ${\varvec{u}}$ denote the density and velocity, respectively, ${\varvec{r}}$ indicates the trajectory of the fluid, and $P$, ${\varvec{\tau}}$, and ${\varvec{g}}$ denote the pressure, deviatoric shear stress tensor, and body force, respectively. The fluid pressure is determined by the equation of state, which can be expressed as:

6 $$\begin{array}{*{20}c} {P = C_{s}^{2} \left( {\rho - \rho_{0} } \right) + P_{0} ,} \\ \end{array}$$

where ${C}_{s}$ is the numerical sound speed, ${\rho }_{0}$ is the reference density, and ${P}_{0}$ is the background pressure.

### Numerical procedure

#### *δ*-*plus*-SPH model

SPH has great advantage when it comes to problems of free surface, deformable boundaries and large-scale deformations, so this method is increasingly popular for studying the behavior of large deformation and post-failure of geomaterial. Based on SPH, we developed the in-house code GeoSPH that has been widely used to study geomaterial flow disasters including the flow-like landslides^11^, fluid–structure interaction^12^ and submarine debris flow^19^, etc. Therefore, this in-house code is utilized in this paper to study the interaction between viscous debris flows and check dams with bottom outlets.

In SPH, the debris flow is discretized by a set of particles. These particles move with the velocity of the fluid and carry physical properties such as the density, mass, and pressure. The physical properties of each particle are calculated through an interpolation process. A function $f\left({{\varvec{r}}}_{i}\right)$ and its derivation $\nabla f\left({{\varvec{r}}}_{i}\right)$ can be calculated as:

7 $$\begin{array}{*{20}c} {f\left( {{\varvec{r}}_{i} } \right) \approx \mathop \sum \limits_{j} f\left( {{\varvec{r}}_{j} } \right)W\left( {{\varvec{r}}_{i} - {\varvec{r}}_{j} ,h} \right)V_{j} ,} \\ \end{array}$$

8 $$\begin{array}{*{20}c} {\nabla f\left( {{\varvec{r}}_{i} } \right) \approx \mathop \sum \limits_{j} f\left( {{\varvec{r}}_{j} } \right)\nabla_{i} W_{ij} V_{j} ,} \\ \end{array}$$

9 $$\begin{array}{*{20}c} {\nabla_{i} W_{ij} = \frac{{{\varvec{r}}_{i} - {\varvec{r}}_{j} }}{{r_{ij} }}\frac{{\partial W_{ij} }}{{\partial r_{ij} }},} \\ \end{array}$$

where ${{\varvec{r}}}_{i}$ is the position of particle $i$, $W\left({{\varvec{r}}}_{i}-{{\varvec{r}}}_{j},h\right)$ is the kernel function, and $h$ is the smoothing length. The subscript $j$ indicates neighboring particles within the support domain. ${r}_{ij}$ is the distance between particles *i* and *j* and $V$ is the volume of an individual particle. In the present work, a Wendland kernel function^20^ is applied:

10 $$\begin{array}{*{20}c} {W_{ij} = W\left( {{\varvec{r}}_{i} - {\varvec{r}}_{j} ,h} \right) = \alpha_{D} \left( {1 - \frac{q}{2}} \right)^{4} \left( {2q + 1} \right) \quad 0 \le q \le 2,} \\ \end{array}$$

where ${\alpha }_{D}=\frac{7}{4\pi {h}^{2}}$ for two-dimension problems and $q=\frac{{r}_{ij}}{h}$.

The weakly compressible SPH method suffers from pressure oscillation. The *δ*-SPH model improves the evaluation of the pressure field by introducing an artificial diffusive term. In addition, a nonuniform particle distribution may have a negative impact on the stability of the SPH method^21^. The particle shifting technique (PST) is a popular method for maintaining the uniformity of the particles^22^. By combining the advantages of the *δ*-SPH scheme and the PST, Sun et al.^23^ proposed the *δ*-*Plus*-SPH scheme. Under the *δ*-*Plus*-SPH scheme, the discrete form of Eq. (5) can be expressed as:

11 $$\left\{ \begin{aligned} \frac{{d\rho_{i} }}{dt} & = - \rho_{i} \mathop \sum \limits_{j} \left[ {\left( {{\varvec{u}}_{j} + \delta {\varvec{u}}_{j} } \right) - \left( {{\varvec{u}}_{i} + \delta {\varvec{u}}_{i} } \right)} \right] \cdot \nabla_{i} W_{ij} V_{j} + \mathop \sum \limits_{j} \left( {\rho_{j} \delta {\varvec{u}}_{j} + \rho_{i} \delta {\varvec{u}}_{i} } \right) \cdot \nabla_{i} W_{ij} V_{j} + \delta hc_{s} {\mathcal{D}}_{i} , \\ \frac{{d{\varvec{u}}_{i} }}{dt} & { = } - \frac{1}{{\rho_{i} }}\mathop \sum \limits_{j} \left( {P_{i} + P_{j} } \right)\nabla_{i} W_{ij} V_{j} + \frac{{\rho_{0} }}{{\rho_{i} }}\alpha \mathop \sum \limits_{j} \frac{{2\eta_{i} \eta_{j} }}{{\eta_{i} + \eta_{j} }}\frac{{\left( {{\varvec{r}}_{i} - {\varvec{r}}_{j} } \right) \cdot \nabla W_{ij} V_{j} }}{{r_{ij}^{2} }}\left( {{\varvec{u}}_{i} - {\varvec{u}}_{j} } \right) \\ & \quad + \mathop \sum \limits_{j} \left( {{\varvec{u}}_{j} \otimes \delta {\varvec{u}}_{j} + {\varvec{u}}_{i} \otimes \delta {\varvec{u}}_{i} } \right) \cdot \nabla_{i} W_{ij} V_{j} - {\varvec{u}}_{i} \mathop \sum \limits_{j} \left( {\delta {\varvec{u}}_{j} - \delta {\varvec{u}}_{i} } \right) \cdot \nabla_{i} W_{ij} V_{j} { + }{\varvec{g}}, \\ \frac{{d{\varvec{r}}_{i} }}{dt} & = {\varvec{u}}_{i} + \delta {\varvec{u}}_{i} , \\ \end{aligned} \right.$$

where the coefficient of the viscous term $\alpha$ is equal to 8 in two-dimensional problems and equal to 10 in three-dimensional problems^24^. The diffusion term ${\mathcal{D}}_{i}$ removes the pressure noise, and the recommended value of the diffusion coefficient $\delta$ is 0.1. ${\mathcal{D}}_{i}$ can be expressed in the following form^25^:

12 $$\begin{array}{*{20}c} {{\mathcal{D}}_{i} = \mathop \sum \limits_{j} \left[ {2\left( {\rho_{i} - \rho_{j} } \right) - \left( {\nabla \left( \rho \right)_{i}^{L} + \nabla \left( \rho \right)_{j}^{L} } \right) \cdot \left( {{\varvec{r}}_{j} - {\varvec{r}}_{i} } \right)} \right]\frac{{{\varvec{r}}_{j} - {\varvec{r}}_{i} }}{{r_{ij} }}V_{j} ,} \\ \end{array}$$

where ${\langle \nabla \left(\rho \right)\rangle }_{i}^{L}$ is the gradient of the density, which is calculated through the renormalized gradient form as^26^:

13 $$\begin{array}{*{20}c} {\left\{ {\begin{array}{*{20}l} {\nabla \left( \rho \right)_{i}^{L} : = \mathop \sum \limits_{j} \left( {\rho_{j} - \rho_{i} } \right){\varvec{L}}_{i} \cdot \nabla_{i} W_{ij} V_{j} ,} \hfill \\ {{\varvec{L}}_{i} = \left[ {\mathop \sum \limits_{j} \left( {{\varvec{r}}_{j} - {\varvec{r}}_{i} } \right) \otimes \nabla_{i} W_{ij} V_{j} } \right]^{ - 1} .} \hfill \\ \end{array} } \right.} \\ \end{array}$$

In the Herschel–Bulkley model, a “cut off” shear rate ${\dot{\gamma }}_{cutoff}$ is used to avoid a singular viscosity occurring at zero shear rate. Therefore, the apparent viscosity of particle *i*, ${\eta }_{i}$, can be expressed as:

14 $$\begin{array}{*{20}c} {\eta_{i} = \left\{ {\begin{array}{*{20}l} {\eta_{cutoff}\quad if \dot{\gamma }_{i} < \dot{\gamma }_{cutoff} ,} \hfill \\ {\frac{{\tau_{y} }}{{\dot{\gamma }_{i} }} + K\dot{\gamma }_{i}^{N - 1} \quad if \dot{\gamma }_{i} \ge \dot{\gamma }_{cutoff} .} \hfill \\ \end{array} } \right.} \\ \end{array}$$

where ${\tau }_{y}$ is constant for viscous debris flows, and ${\tau }_{y}$ is equal to $ptan\phi +c$ for landslides^11^, $\phi$ is the frictional angle and $c$ is the cohesion.

The rate-of-strain tensor ${\varvec{S}}$ is calculated as:

15 $$\begin{array}{*{20}c} {S = \frac{1}{2}\mathop \sum \limits_{j} \left[ {\left( {{\varvec{u}}_{j} - {\varvec{u}}_{i} } \right) \otimes \left( {{\varvec{L}}_{i} \cdot \nabla_{i} W_{ij} } \right) + \left( {{\varvec{L}}_{i} \cdot \nabla_{i} W_{ij} } \right) \otimes \left( {{\varvec{u}}_{i} - {\varvec{u}}_{j} } \right)} \right]V_{j} .} \\ \end{array}$$

In Eq. (11), $\delta {\varvec{u}}$ is the arbitrary velocity calculated by PST to maintain a uniform particle configuration. The shifting velocity $\delta {\overline{{\varvec{u}}} }_{i}$ can be written as^24^:

16 $$\begin{array}{*{20}c} {\delta \overline{\user2{u}}_{i} = - U_{max} \left( {2h} \right)\sum \left[ {1 + R\left( {\frac{{W_{ij} }}{{W\left( {\Delta x} \right)}}} \right)^{n} } \right]\nabla_{i} W_{ij} V_{j} ,} \\ \end{array}$$

where $R$ and $n$ are set to 0.2 and 4, respectively^24^. dx is the initial particle distance, and ${U}_{max}$ is the maximum velocity, $\Delta x$ is the initial particle distance. To prevent $\delta {\overline{{\varvec{u}}} }_{i}$ from becoming too large and maintain a consistent kinematic boundary condition, the velocity deviation $\delta {\varvec{u}}$ is calculated as^25^:

17 $$\begin{array}{*{20}c} {\delta {\varvec{u}}_{i} = \left\{ {\begin{array}{*{20}l} {0 \quad if \lambda_{i} < 0.55,} \hfill \\ {\left( {{\varvec{I}} - {\varvec{n}}_{i} \otimes {\varvec{n}}_{i} } \right)\delta {\varvec{u}}_{i}^{*} \quad if 0.55 \le \lambda_{i} \le 0.90\, and\, \delta {\varvec{u}}_{i}^{*} \cdot {\varvec{n}}_{i} \ge 0,} \hfill \\ {\delta {\varvec{u}}_{i}^{*} \,\quad if\, 0.55 \le \lambda_{i} \le 0.90 \,and \,\delta {\varvec{u}}_{i}^{*} \cdot {\varvec{n}}_{i} < 0,} \hfill \\ {\delta {\varvec{u}}_{i}^{*} \, \quad if\, \lambda_{i} > 0.90,} \hfill \\ \end{array} } \right.} \\ \end{array}$$

where ${{\varvec{u}}}_{i}^{*}=\mathrm{min}\left(\Vert \delta {\overline{{\varvec{u}}} }_{i}\Vert ,\frac{{U}_{max}}{2}\right)\frac{\delta {\overline{{\varvec{u}}} }_{i}}{\Vert \delta {\overline{{\varvec{u}}} }_{i}\Vert }$, ${\lambda }_{i}$ is the minimum eigenvalue of the tensor ${\varvec{B}}_{i} = \left[ {\sum\nolimits_{j \in \chi } {\left( {{\varvec{r}}_{j} - {\varvec{r}}_{i} } \right) \otimes \nabla_{i} W_{ij} V_{j} } } \right]$, ${{\varvec{n}}}_{i}$ is the normal vector to the free surface of particle $i$. More details of the PST can be found in the research by Sun et al.^23^.

In this study, the fixed ghost particle technique^27^ is applied to model the solid boundaries. That is, the solid boundaries are discretized by fixed ghost particles, and there is a corresponding interpolation particle in the fluid domain for each fixed ghost particle. The physical quantities of the fixed ghost particles are calculated based on the moving least-squares (MLS) interpolation of fluid particles. To prevent particle penetration, the pressure of the fixed ghost particles can be expressed as:

18 $$\begin{array}{*{20}c} {P_{G} = \mathop \sum \limits_{j} P_{j} W^{MLS} \left( {{\varvec{r}}_{j} } \right)V_{j} + d\rho_{f} {\varvec{n}} \cdot {\varvec{g}},} \\ \end{array}$$

where $d$ indicates the distance between the ghost particle and the corresponding interpolation particle, ${\varvec{n}}$ denotes the direction vector between the ghost particle and the corresponding interpolation particle, ${\rho }_{f}$ is the reference density of the denser fluid, and ${W}^{MLS}$ is the MLS kernel, which can be calculated as^27^:

19 $$\begin{array}{*{20}c} {\left\{ {\begin{array}{*{20}l} {W^{MLS} \left( {{\varvec{r}}_{j} } \right) = {\varvec{M}}_{i}^{ - 1} {\varvec{e}}_{1} \cdot {\varvec{b}}_{ij} W\left( {{\varvec{r}}_{j} } \right),} \hfill \\ {{\varvec{M}}_{i} = \mathop \sum \limits_{j} {\varvec{b}}_{ij} \otimes {\varvec{b}}_{ij} W\left( {{\varvec{r}}_{j} } \right)V_{j} ,} \hfill \\ {{\varvec{b}}_{ij}^{T} = \left[ {1, \left( {x_{j} - x_{i} } \right),\left( {y_{j} - y_{i} } \right)} \right],} \hfill \\ {{\varvec{e}}_{1}^{T} = \left[ {1,0,0} \right].} \hfill \\ \end{array} } \right.} \\ \end{array}$$

To enforce the no-slip condition, the velocity of the fixed ghost particles can be calculated as follows:

20 $$\begin{array}{*{20}c} {{\varvec{u}}_{G} = - \mathop \sum \limits_{j} {\varvec{u}}_{j} W^{MLS} \left( {{\varvec{r}}_{j} } \right)V_{j} .} \\ \end{array}$$

The 4th-order Runge–Kutta scheme is applied in this study. A GPU parallelization technique is implemented to accelerate the simulations.

#### Model validation

**Figure 2:**
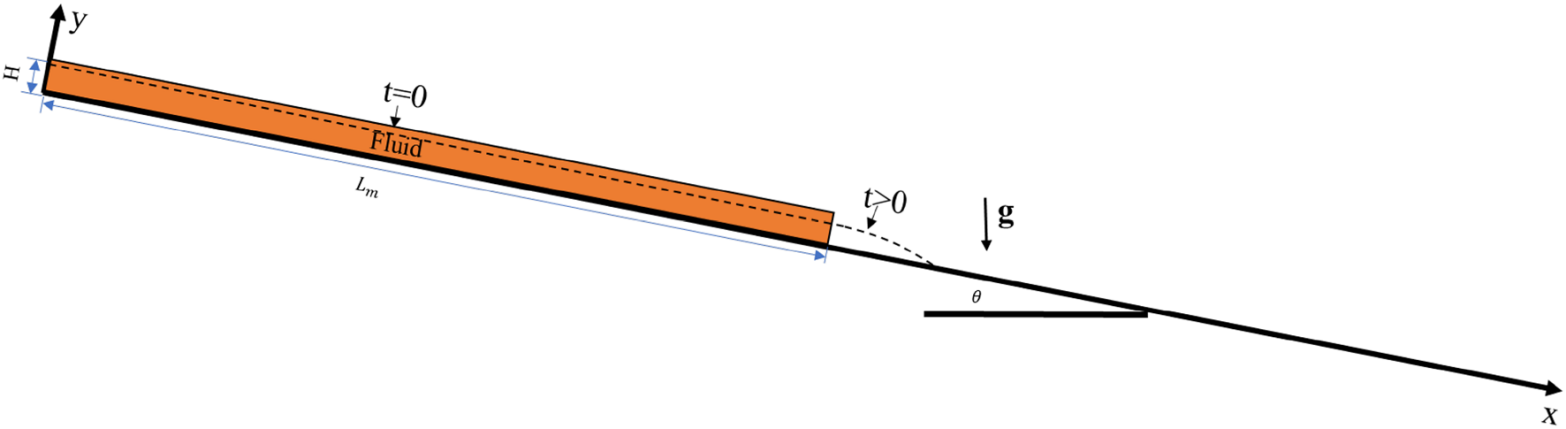
Geometrical configuration of the dam break test.

Komatina and Jovanovic^28^ conducted a series of dam break tests to study the steady and unsteady free surface flow of non-Newtonian fluids, the basic configuration of the dam break tests is shown in Fig. 2. In their study, a fluid (${L}_{m}=2.0$ m and H = 0.1 m) slides down a 0.1% slope. The unit weight of the test fluid is 12.0 kNm^−3^. The values of ${\tau }_{y}$, $K$, and $N$ for the test fluid are 25 Pa, 0.07 Pa s^*N*^, and 1.0, respectively. The propagation of the leading edges was recorded in their test, and no rigid barrier was installed in front of the test fluid.

In present work, the “cutoff” shear rate ${\dot{\gamma }}_{cutoff}$ is adopted to avoid a singular viscosity occurring at zero shear rate. Parametric analysis conducted by Manenti et al.^29^ shows that a suitable maximum viscosity or “cutoff” shear rate will save computational time while ensuring the numerical accuracy. Thus, the value of ${\dot{\gamma }}_{cutoff}$ should be evaluated through a convergence analysis.

To study the influence of ${\dot{\gamma }}_{cutoff}$**,** the initial spacing of the particles dx is set as 5 mm, and there are 8000 fluid particles simulating the test fluid. Five runs are conducted that the ${\dot{\gamma }}_{cutoff}$ varies from 0.001 to 10 s^−1^.

**Figure 3:**
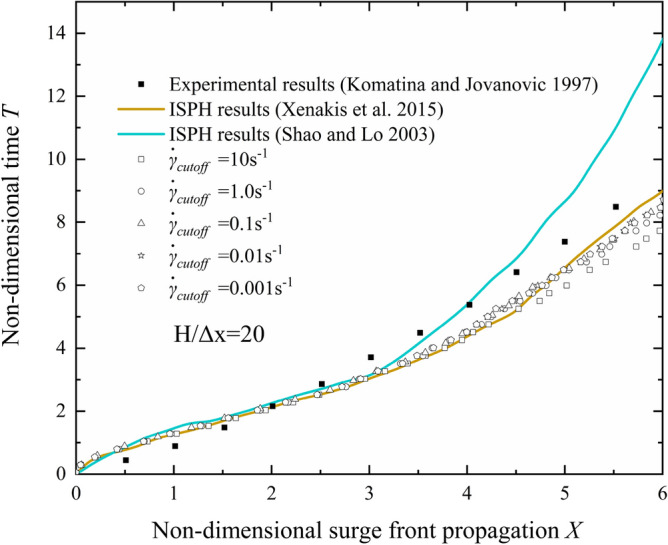
Effect of “cutoff” shear rate on surge front propagation.

Figure 3 compares the experimental results and simulation results, where the dimensionless surge front propagation is calculated as $X=(x-L)/H$ and the dimensionless time is calculated as $T=t{(g/H)}^{0.5}$. As shown in Fig. 3, all the simulation results of surge front are slower than experimental results when $T<2.0$. This phenomenon can be attributed to the non-slip boundary that is widely used in the SPH method. The particles at the bottom of the leading edge are hindered by the no-slip boundary, which results in the simulation results are slower than experimental results when $T<2.0$.

As shown in Fig. 3, the value of ${\dot{\gamma }}_{cutoff}$ have obvious influence on the surge front propagation when $T>2.0$. As ${\dot{\gamma }}_{cutoff}$ decreases, the propagation of the leading edge of the fluid becomes progressively slower when $T>3.5$. And there is no noticeable variation between surge front propagation curves when ${\dot{\gamma }}_{cutoff}\le 0.1 {s}^{-1}$. Therefore, the ${\dot{\gamma }}_{cutoff}$ will set as 0.1 s^-1^ in subsequent simulations.

Compared with previous numerical results, the current results are very close to those of Xenakis et al.^30^ This is due to the fact that bilinear model adopted by Xenakis et al.^30^ is very similar to the “cut off” shear rate adopted in this work. On the other hand, the cross model adopted by Shao and Lo^31^ will produce a very large apparent viscosity when the fluid is under a small shear rate, which results in a slower propagation for $T>4.0$.

**Figure 4:**
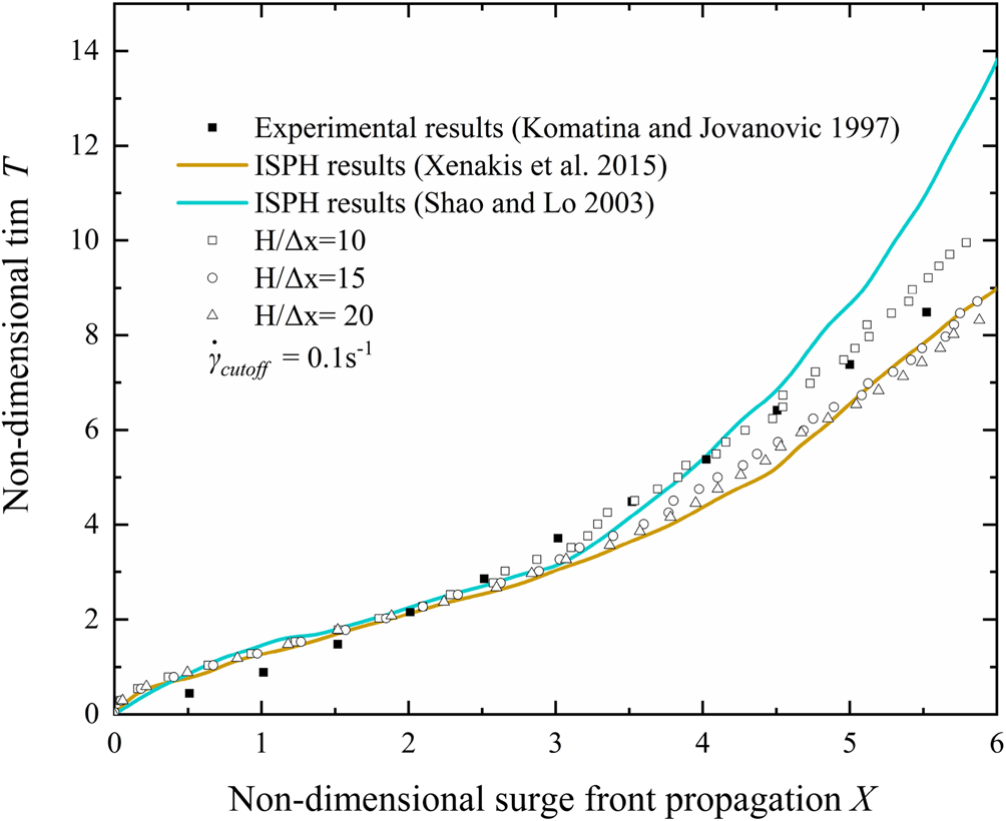
Effect of particle resolution on surge front propagation.

In addition to “cut off” shear rate, particle resolution is another parameter affecting the stability and accuracy of numerical calculation. Three addition runs are conducted that the particle resolution H/$\Delta x$ vary from 10 to 20. As shown in Fig. 4, surge front propagation of the coarsest particle resolution case is much slower than those of finer particle resolution cases. For the coarsest particle resolution case, all particles in the leading edge of the fluid are under the influence of no-slip boundary condition, which leads to a slower propagation of surge front. Thus, to ensure numerical accuracy, the particle resolution in the subsequent simulations is finer than H/$\Delta x$=10.

Manenti et al.^14^ conducted a numerical study on a full-scale rainfall-induced fast shallow landslide occurred in Italy by SPH. This landslide impacted against the wall of a building. Thus, this case is performed to verify the reliability of the proposed SPH method for modeling the free flow and impingement of non-Newtonian fluids. The input parameters of this case are listed in Table 1.

**Table 1:** Summary of the input parameters.

Parameters	Value	Reference
Particle resolution $\Delta x$ (m)	0.10	–
Density $\rho$ (kg/m^3^)	1957	Manenti et al.^14^
Angle of internal friction $\phi$ (°)	24	Manenti et al.^14^
Cohesion *c* (Pa)	0	Manenti et al.^14^
Power law exponent $N$	1	Manenti et al.^14^
Consistency index *K* (Pa∙s^*N*^)	1	–

**Figure 5:**
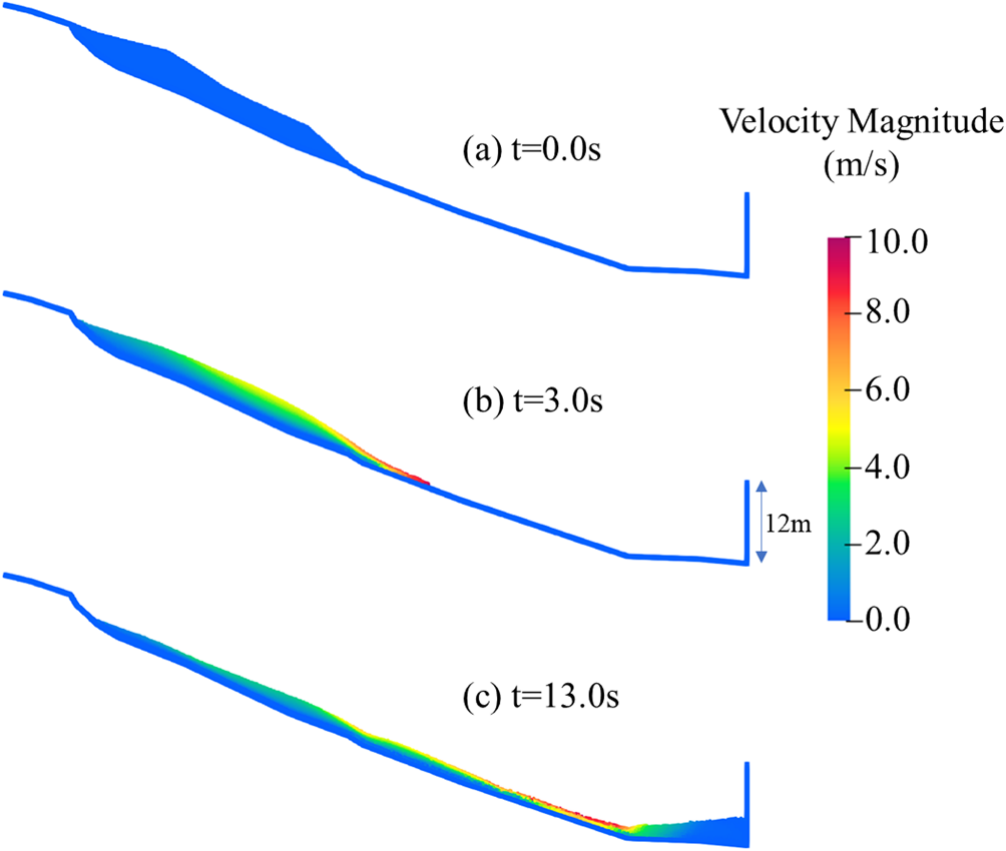
Evolution of the velocity fields at typical instants.

**Figure 6:**
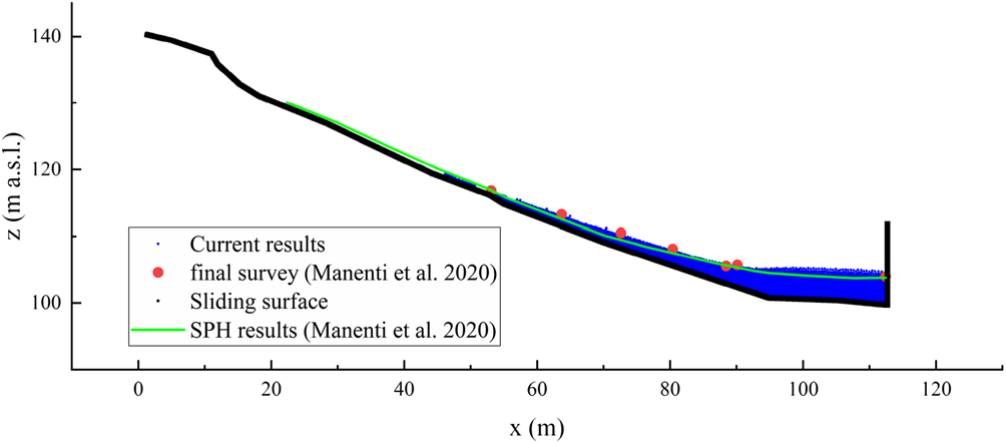
Comparison of final profile at t = 60 s.

Figure 5 shows the evolution of the velocity fields at typical instants. At t = 3.0 s, the landslide front began accelerating under gravity. Then, the landslide impact against the vertical wall. As shown in Fig. 5c, due to the obstructive effect of the wall, the landslide front was decelerated and stopped in front of the wall. The finial profile of the landslide was compared with the on-site survey^14^ and WCSPH computational results^14^, as shown in Fig. 6. The run-up length on the vertical wall is 4.0 m. In this numerical test, the simulated run-up length on the vertical wall is 4.2 m. The results show that the current SPH model can provide a reasonable prediction of the final profile of the landslide.

In general, the proposed SPH model can reasonably describe the propagation and impingement of a non-Newtonian fluid under gravity.

### Test program

In this paper, the influence of check dams with bottom outlets on viscous debris flow mobility is numerically investigated by changing the size of the bottom outlet ${H}_{c}$ and the slope angle *θ*. The numerical test program is listed in Table 2.

**Table 2:** Numerical test program.

Test ID	${H}_{c}$(cm)	*θ* (°)	${h}_{max}$(cm)	${\overline{u} }_{max}$(m/s)	$Fr$	${H}_{j}$(cm)	Jump height predicted by frictionless finite mass model^33^ (cm)	${Q}_{p}$ (m^2^/s)	${Q}_{r}$(m^2^/s)	RE	EB
I15	–	15	6.07	1.44	1.90	–	–	0.759	0.00119	52.293	0
I25	–	25	6.52	2.22	2.92	–	–	0.141	0.00117	32.088	0
I35	–	35	6.94	2.93	3.92	–	–	0.202	0.00109	22.300	0
I15–H0	0.0	15	–	–	1.90	30.5	28.0	0	0	100.0	1
I25–H0	0.0	25	–	–	2.92	64.2	62.0	0	0	100.0	1
I35–H0	0.0	35	–	–	3.92	99.6	113.8	0	0	100.0	1
I15–H1	1.0	15	–	–	1.90	29.6	–	0.003	0.000257	98.479	0.999
I15–H2	2.0	15	–	–	1.90	25.8	–	0.020	0.00175	88.834	0.935
I15–H3	3.0	15	–	–	1.90	22.7	–	0.039	0.00287	74.674	0.736
I15–H4	4.0	15	–	–	1.90	16.8	–	0.054	0.00238	60.809	0.438
I15–H5	5.0	15	–	–	1.90	13.0	–	0.068	0.00128	53.274	0.171
I25–H1	1.0	25	–	–	2.92	61.9	–	0.016	0.00073	96.100	0.994
I25–H2	2.0	25	–	–	2.92	56.3	–	0.0380	0.00387	73.833	0.862
I25–H3	3.0	25	–	–	2.92	51.6	–	0.062	0.00511	47.682	0.649
I25–H4	4.0	25	–	–	2.92	40.2	–	0.087	0.00116	33.128	0.368
I25–H5	5.0	25	–	–	2.92	32.5	–	0.110	0.00131	33.107	0.196
I35–H1	1.0	35	–	–	3.92	96.3	–	0.023	0.00111	93.774	0.991
I35–H2	2.0	35	–	–	3.92	88.4	–	0.050	0.0054	61.704	0.822
I35–H3	3.0	35	–	–	3.92	83.3	–	0.081	0.00791	29.764	0.612
I35–H4	4.0	35	–	–	3.92	73.1	–	0.114	0.00143	24.154	0.399
I35–H5	5.0	35	–	–	3.92	60.6	–	0.140	0.00105	24.049	0.258

As shown in Fig. 1, two monitor sections are set during the whole simulation process of each test to better understand the evolution of debris flow. The monitor section I is set between x = 1.58 m and x = 1.61 m. The evolution of flow depth (*h*) and flow velocity ($\overline{u }$) at check dam installed position (x = 1.60 m) can be calculated based on the following equations:

21 $$\begin{array}{*{20}c} {h = \frac{2}{{N_{p} }}\mathop \sum \limits_{i = 1}^{{N_{p} }} y_{i} ,} \\ \end{array}$$

22 $$\begin{array}{*{20}c} {\overline{u} = \frac{1}{{N_{p} }}\mathop \sum \limits_{i = 1}^{{N_{p} }} u_{i} ,} \\ \end{array}$$

where ${N}_{p}$ is the number of fluid particles within the interaction domain at the monitor section I, ${y}_{i}$ is the y-coordinate of particle *i*, and ${u}_{i}$ is the velocity component of particle *i* in the x-direction*.* To ensure clarity, the maximum flow depth $h$ of free flow tests (I15, I25, I35) is marked as ${h}_{max}$, the maximum flow depth $h$ of check dam installed tests represents the maximum jump height ${H}_{j}$, and specially the maximum flow depth $h$ of closed check dam installed tests (I15-H0, I25-H0, I35-H0) can be marked as ${H}_{j0}$.

The maximum flow depth (${h}_{max}$) and flow velocity (${\overline{u} }_{max}$) are used to calculate the Froude number as:

23 $$\begin{array}{*{20}c} {Fr = \frac{{\overline{u}_{max} }}{{\sqrt {gh_{max} cos\theta } }},} \\ \end{array}$$

where the $cos\theta$ is a gravitational component correction that was proposed by Choi et al.^32^.

The flow kinematics of three control tests (I15, I25, I35) are listed in Table 2. All three control tests are in the supercritical condition when flow front reaches x = 1.60.

The monitor section II is set between x = 1.65 m and x = 1.67 m to investigate the evolution of outflow. The unit width discharge $Q$ and outflow kinetic energy ${E}_{k}$ can be calculated with the following expressions:

24 $$\begin{array}{*{20}c} {Q = \frac{1}{0.02}\mathop \sum \limits_{i = 1}^{{N_{p} }} u_{i} } \\ \end{array}$$

25 $$\begin{array}{*{20}c} {E_{k} = \frac{1}{2}\mathop \sum \limits_{i = 1}^{{N_{p} }} m_{i} v_{i}^{2} ,} \\ \end{array}$$

where ${N}_{p}$ is the number of fluid particles within the interaction domain at the monitor section II, ${m}_{i}$ is the mass of particle $i$, ${u}_{i}$ is the velocity component of particle *i* in the x-direction and ${v}_{i}$ is the velocity magnitude of particle $i$. To ensure clarity, the peak unit width discharge can be marked as ${Q}_{p}$, the unit width discharge at t = 5.0 s can be marked as residual discharge ${Q}_{r}$ and the peak unit width discharge of free flow tests can be marked as ${Q}_{pf}$.

## Results and discussion

### Flow pattern

**Figure 7:**
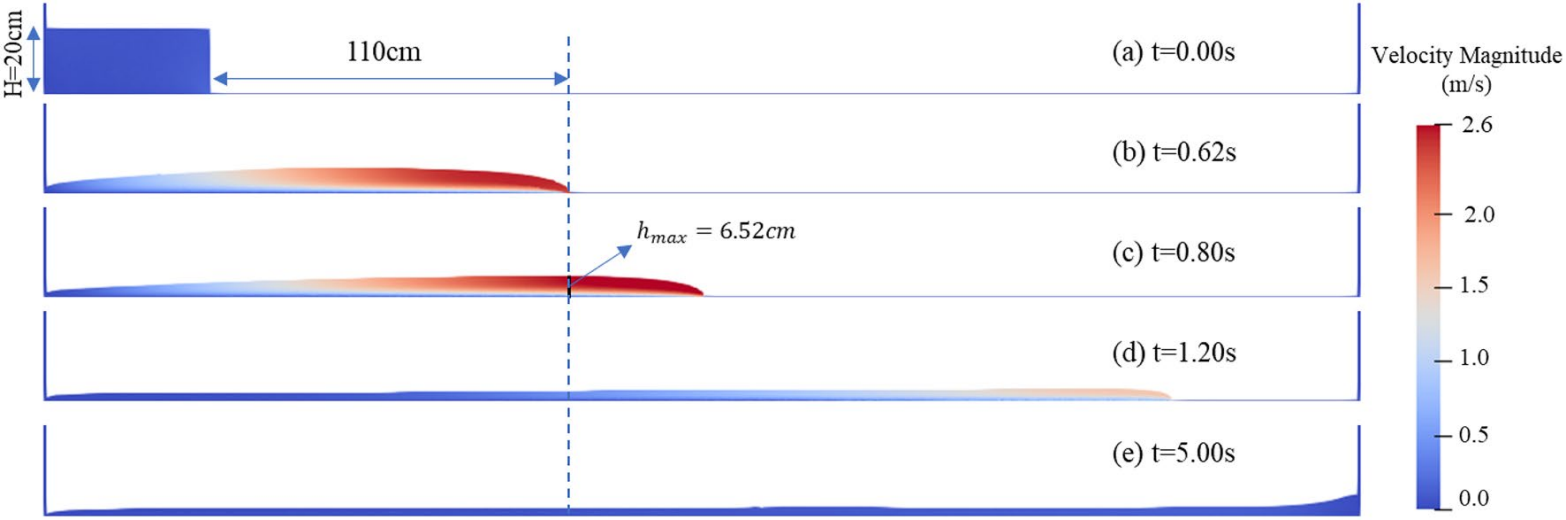
Snapshots of test I25 at typical instants: (**a**) t = 0.00 s; (**b**) t = 0.62 s; (**c**) t = 0.80 s; (**d**) t = 1.20 s; (**e**) t = 5.00 s. (${h}_{max}$=6.52 cm, ${\overline{u} }_{max}$=2.22, $Fr$=2.92)*.* (The high-resolution version of this figure please refer to Supplementary Fig. S1).

Figure 7 shows a series of snapshots of free flow for the test I25. As it descends the flume, the debris flow is accelerated and elongated by gravity. At 0.62 s after the trigger gate opens, a flow front with an approach velocity of 2.22 m/s reaches x = 1.60 m. At t = 0.8 s, the flow depth at x = 1.60 m reaches its maximum value ${h}_{max}$. Then the flow is decelerated and nearly stalled by the influence of no-slip boundary.

**Figure 8:**
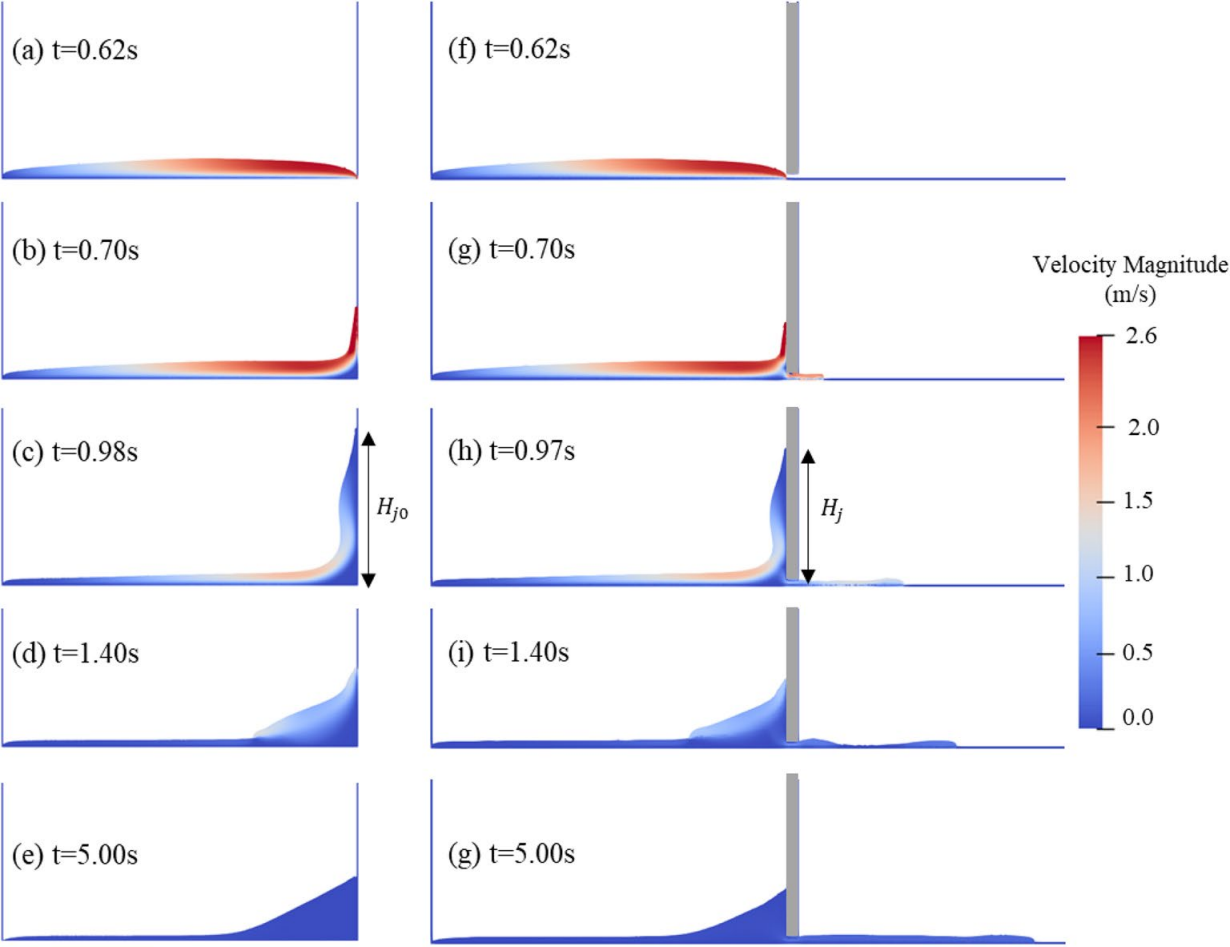
Comparisons between flow fields of test I25-H0 and test I25-H2: (**a**–**e**) flow fields of test I25-H0, (**f**–**g**) flow fields of test I25-H2. (${H}_{j0}$ = 64.2 cm, ${H}_{j}$=56.3 cm).

Figure 8 shows comparisons between flow fields of test I25-H0 and test I25-H2. As shown in Fig. 8b, all the viscos debris flow is blocked by the closed check dam and a distinct upward jet can be observed. On the other hand, the front of the debris flow is blocked by the check dam and divided into upward and downstream jets, as shown in Fig. 8g.The flow pattern of the upward jet is similar to the run-up mechanism described by Choi et al.^34^. The upward jet continues to run up and the jump height continues to increase after the flow front impacts the check dam. Due to the existence of the bottom outlet, test I25-H2 reaches the maximum value of jump height a little earlier than test I25-H0. And the maximum jump height of I25-H2 is obviously lower than that of test I25-H0, as shown in Fig. 8c,h. The run-up process ceases when the maximum jump height is attained. The upward jet then begins to roll back to the flume base, as shown in Fig. 8d,i. The kinetic energy of the upward jet is dissipated by these processes. The velocity of the debris flow behind the check dam decreases drastically. The debris flow has come to a near-rest state 5.0 s after the trigger gate opened.

**Figure 9:**
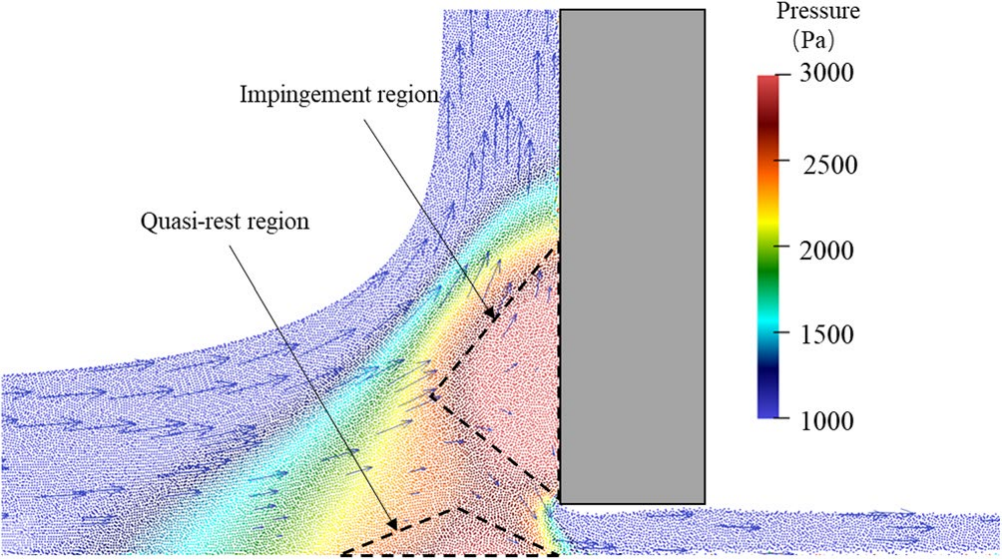
Pressure field and velocity vectors near the bottom outlet of test I25-H2 at t = 0.8 s.

The bottom outlet increases the complexity of the debris flow dynamic process compared to the traditional dam break free flow problem and closed check dam cases. Figure 9 shows that the impingement of the debris flow on the solid surface causes an increase in fluid pressure and significant adverse pressure gradients in the impingement region. The flow direction of the incoming jet changes dramatically in the impingement region. Figure 9 also shows that part of the incoming jet flows up along the wall and another part flows toward the bottom outlet. The adverse pressure gradients hinder the debris flow near the bottom of the check dam. An inverse pressure gradient causes the debris flow near the flume base to lose its forward momentum and form a quasi-rest region. This quasi-rest region acts like a wedge, leading to the upward movement of the subsequent incoming flow. A narrow pathway forms between the impingement region and the quasi-rest region. After flowing through this pathway, the pressure of the debris flow gradually decreases.

### Effect of bottom height on jump height

**Figure 10:**
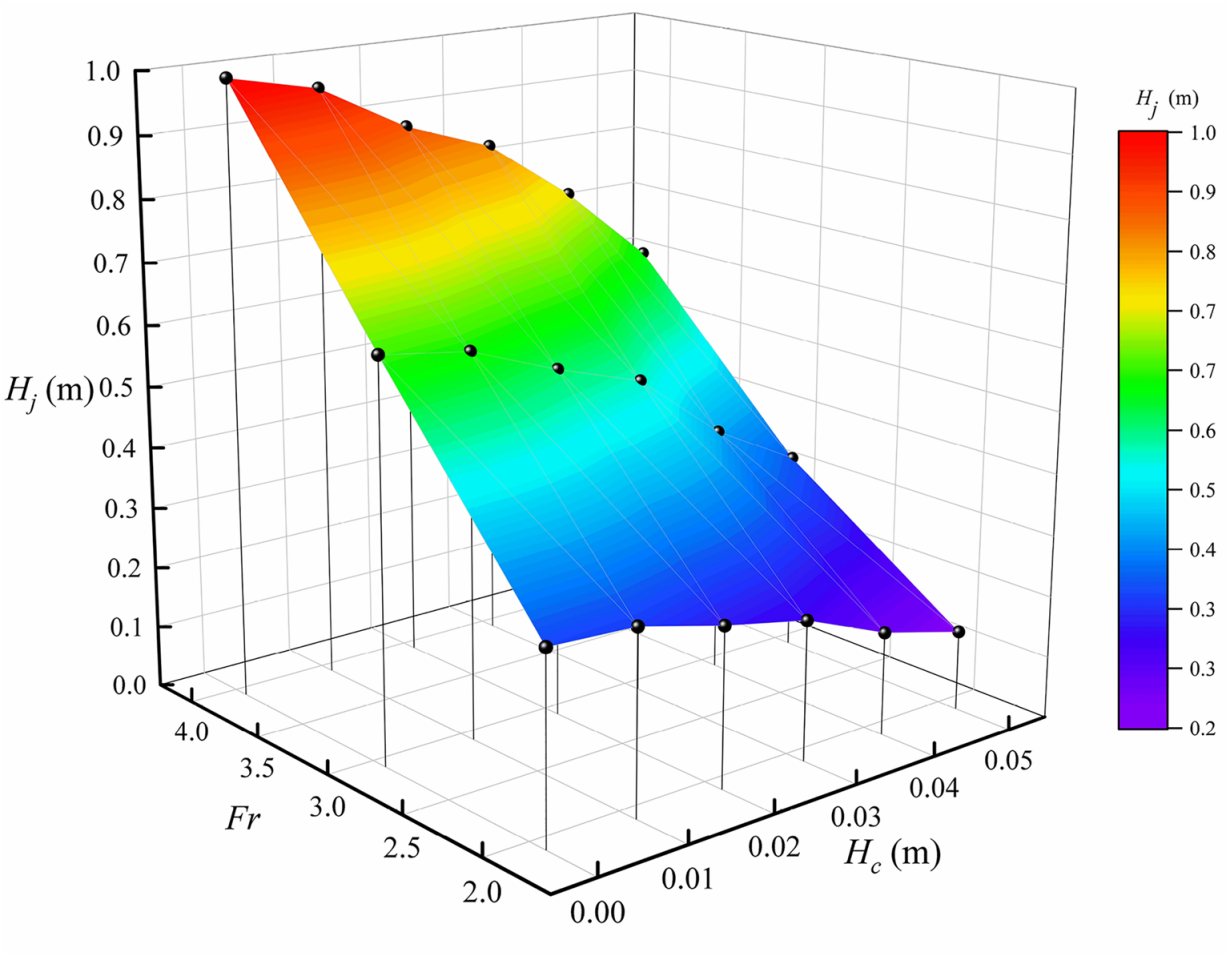
Jump height at different Froude numbers with different bottom outlet sizes.

Show as Fig. 8, after the flow front impact on the check dam, the debris flow runs up and finally reaches the jump height (${H}_{j}$). In designing a check dam, it is important to estimate the jump height (${H}_{j}$) to prevent overtopping and damage of the protective structures^33,35^. In the present study, we monitored the jump height of check dam with different opening sizes at different Froude number, as shown in Fig. 10.

**Figure 11:**
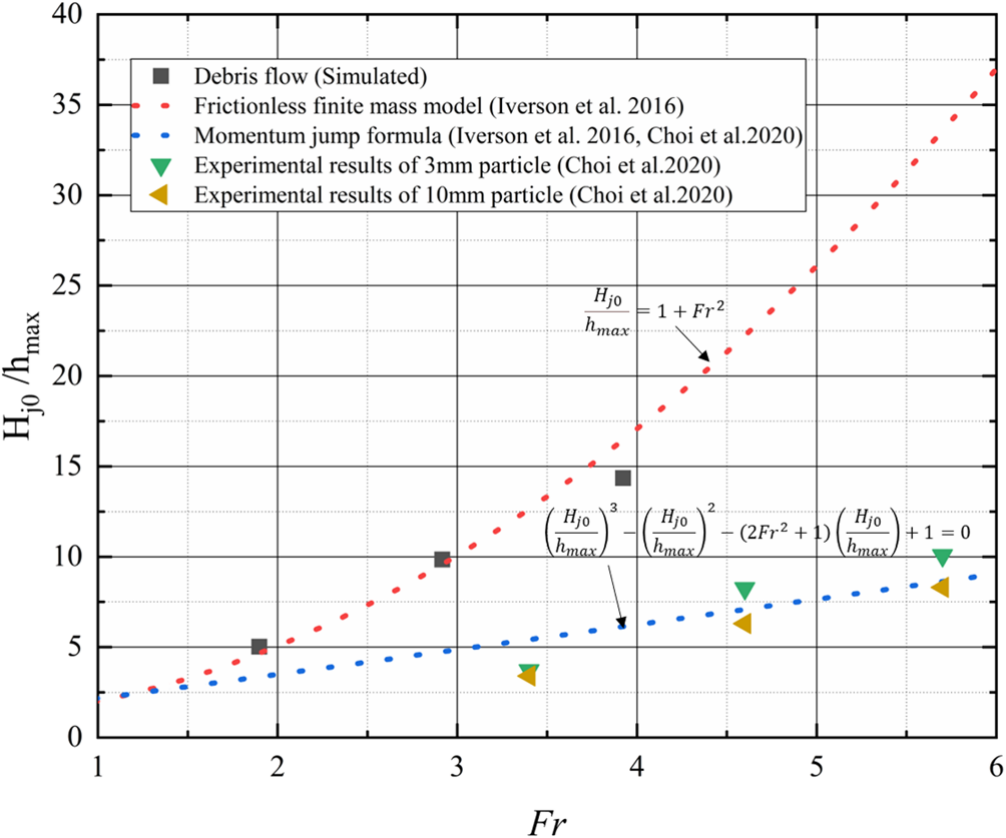
Comparison of simulated peak jump height of closed check dam with experimental results and results of analytical models.

Previous studies have shown that the jump height of closed check dam (${H}_{j0}$) increases with increasing Froude number. The numerical results also show a similar pattern, as shown in Fig. 10. Different analytical models have been proposed to predict the jump height of closed check dams^33,36^. Figure 11 compares the simulated peak jump height of a closed check dam with experimental results conducted by Choi et al.^7^ and results of two classic analytical models. As shown in Fig. 11, there is obvious discrepancy between the simulated peak jump heights and experimental results.And the simulated peak jump heights are close to the predicted value given by the frictionless finite mass model^33^, and the experimental results are close to the predicted value given by momentum jump formula^7,33^.

This phenomenon is in accordance with the different run up mechanisms between viscous debris flow and dry granular flow. For viscous debris flows, upward flow jet is governed by run-up mechanism described by Choi et al.^34^. In viscous debris flows, the stress is dominated by viscoplastic stress^10^ and the energy loss is relatively small during the impact process. Meanwhile, the frictionless finite mass model is proposed based on the energy balance principle. Thus, the results of frictionless finite mass model are very close to the simulated results. On the other hand, dry granular flow consists of frictional material with shear strength and impact process is governed by the pile-up described by Choi et al.^34^. The pileup mechanism is similar to the mechanism for the momentum jump formula^7,33^. Thus, the momentum jump formula can produce good results for dry granular flow.

**Figure 12:**
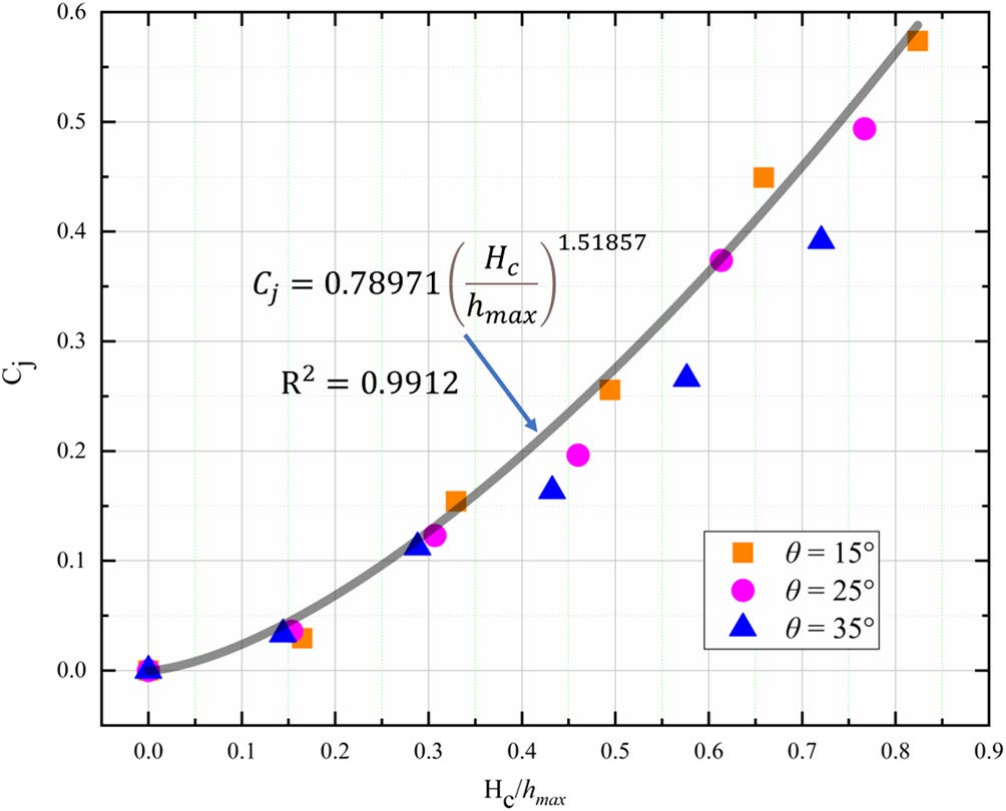
Dependence of peak jump height decay coefficient (${C}_{j}$) on normalized height of the bottom outlet (${H}_{c}/{h}_{max}$) and slope angles.

Because of the transport of kinetic energy by the bottom outlet, the jump height of opening check dam ${H}_{j}$ is usually smaller than the jump height of closed check dam ${H}_{j0}$ for a given Froude number, as shown in Fig. 12. In this study, we define the jump height decay coefficient ${C}_{j}$ as:

26 $$\begin{array}{*{20}c} {C_{j} = 1 - \frac{{H_{j} }}{{H_{j0} }}.} \\ \end{array}$$

The dependence of ${C}_{j}$ on the normalized height of the bottom outlet (${H}_{c}/{h}_{max}$) and slope angle is plotted in Fig. 12. Compared with the slope angle, ${H}_{c}/{h}_{max}$ has a greater influence on ${C}_{j}$. And the ${C}_{j}$ increases with ${H}_{c}/{h}_{max}$ with a power function. Thus, the power function is used to fit the relationship between ${C}_{j}$ and ${H}_{c}/{h}_{max}$. And the best fitting curve between ${C}_{j}$ and ${H}_{c}/{h}_{max}$ can be expressed as:

27 $$\begin{array}{*{20}c} {C_{j} = 0.78971\left( {\frac{{H_{c} }}{{h_{max} }}} \right)^{1.51857} .} \\ \end{array}$$

### Effect of bottom height on outflow rate and retention efficiency

**Figure 13:**
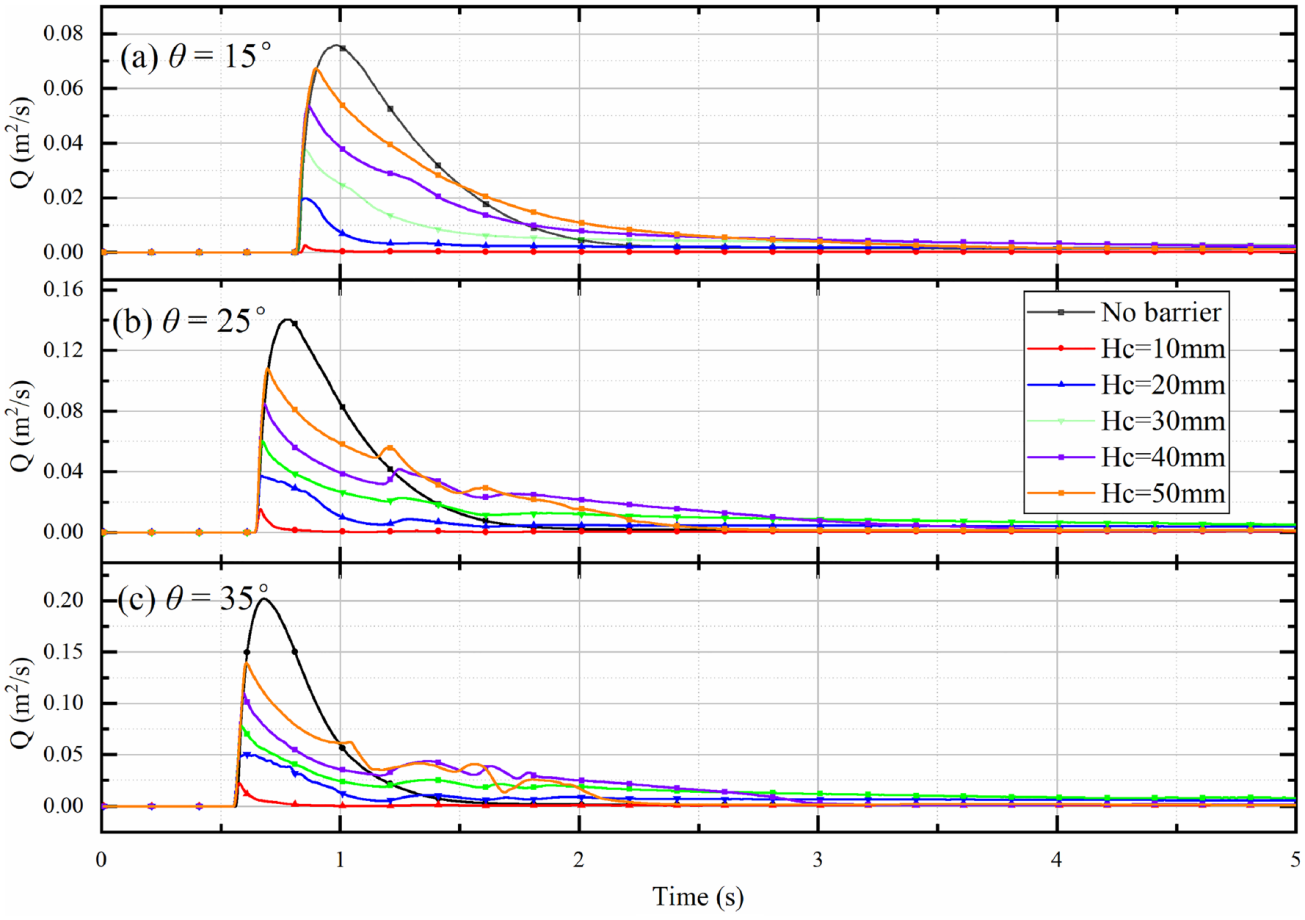
Evolution of outflow rates at different slope angles with different bottom outlet sizes.

One of the main functions of an open check dam is to regulate the discharge of the debris flow. The outflow rates (Q) at $x=1.65 \mathrm{m}$ for different slope angles with different bottom outlet sizes are summarized in Fig. 13. The outflow rates in the case of a free flow (no barrier) are also plotted in Fig. 13 for references. For a free flow when $\theta =15^\circ$, the flow leading edge reaches $x=1.65 \mathrm{m}$ at 0.79 s. The flow rate reaches the peak value (Q_pf_) soon after, at 0.99 s. After the peak value, the flow rate in the free flow case decreases at a relatively slow rate, and ultimately reaches a residual state after around 3.0 s.

**Figure 14:**
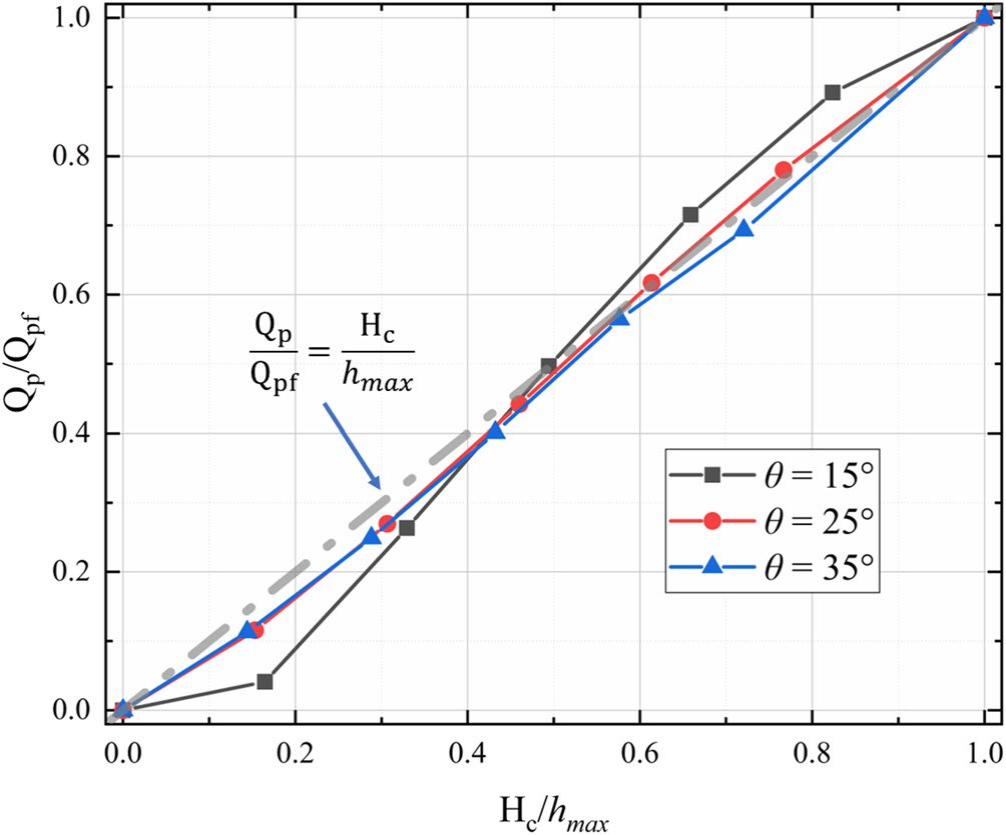
Relationship between normalized peak outflow rate (Q_p_/Q_pf_) and normalized bottom outlet height (${H}_{c}/{h}_{max}$) for various slope angles.

As shown in Fig. 13, the check dam has a significant impact on the peak outflow rate. A larger bottom outlet size results in a larger peak outflow rate. To better understand the influence of the bottom outlet on the peak outflow rate, the dependence of the normalized peak outflow rate (Q_p_/Q_pf_) on the normalized height of the bottom outlet (${H}_{c}/{h}_{max}$) and the slope angle is plotted in Fig. 14. There is a strong positive correlation between ${H}_{c}/{h}_{max}$ and Q_p_/Q_pf_. For relatively small normalized bottom outlet heights ($\frac{{H}_{c}}{{h}_{max}}<0.5$), Q_p_/Q_pf_ is less than the corresponding ${H}_{c}/{h}_{max}$ because of the drag force produced by the bottom of the check dam and the flume base. For larger values of the normalized bottom outlet height ($\frac{{H}_{c}}{{h}_{max}}>0.5$), the effect of the drag force is relatively small compared with the energy carried by the outflow, resulting in Q_p_/Q_of_ being close to the corresponding ${H}_{c}/{h}_{max}$.

**Figure 15:**
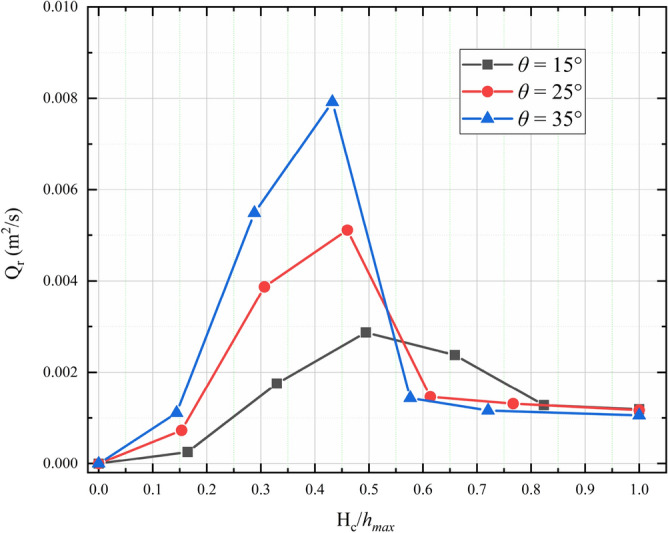
Dependence of outflow rate after 5.0 s on normalized bottom outlet height (${H}_{c}/{h}_{max}$) for various slope angles.

Figure 15 shows the outflow rate after 5.0 s (Q_r_) for various normalized bottom outlet heights (${H}_{c}/{h}_{max}$) and slope angles. For relatively small values of the normalized bottom outlet height ($\frac{{H}_{c}}{{h}_{max}}<0.5$), Q_r_ increases as the slope angle becomes steeper and ${H}_{c}/{h}_{max}$ increases. However, Q_r_ decreases with increasing normalized bottom outlet height when $\frac{{H}_{c}}{{h}_{max}}>0.5$. A steeper slope produces a faster decrease in Q_r_. When $\frac{{H}_{c}}{{h}_{max}}>0.8$, Q_r_ for the open check dam is very close to Q_r_ in the case of free flow.

Before the construction of the opening check dam, the trapping objective should be determined by the designer depending on the stream features, desilting period and the design life^3^. As the debris flow moves away from the bottom outlet, the volume of the debris flow remaining upstream of the check dam decreases. Thus, designers should determine the size of bottom outlet to meet the trapping objective. The retention efficiency (*RE*) is a widely used indicator that can quantitatively describe the trapping objective^8^, which can be expressed as:

28 $$\begin{array}{*{20}c} {RE = \frac{{m_{{{\text{retention}}}} }}{{m_{{{\text{total}}}} }} \times 100,} \\ \end{array}$$

where ${m}_{\mathrm{retention}}$ is the mass of the debris flow remaining upstream of the check dam and ${m}_{\mathrm{total}}$ is the total debris mass.

**Figure 16:**
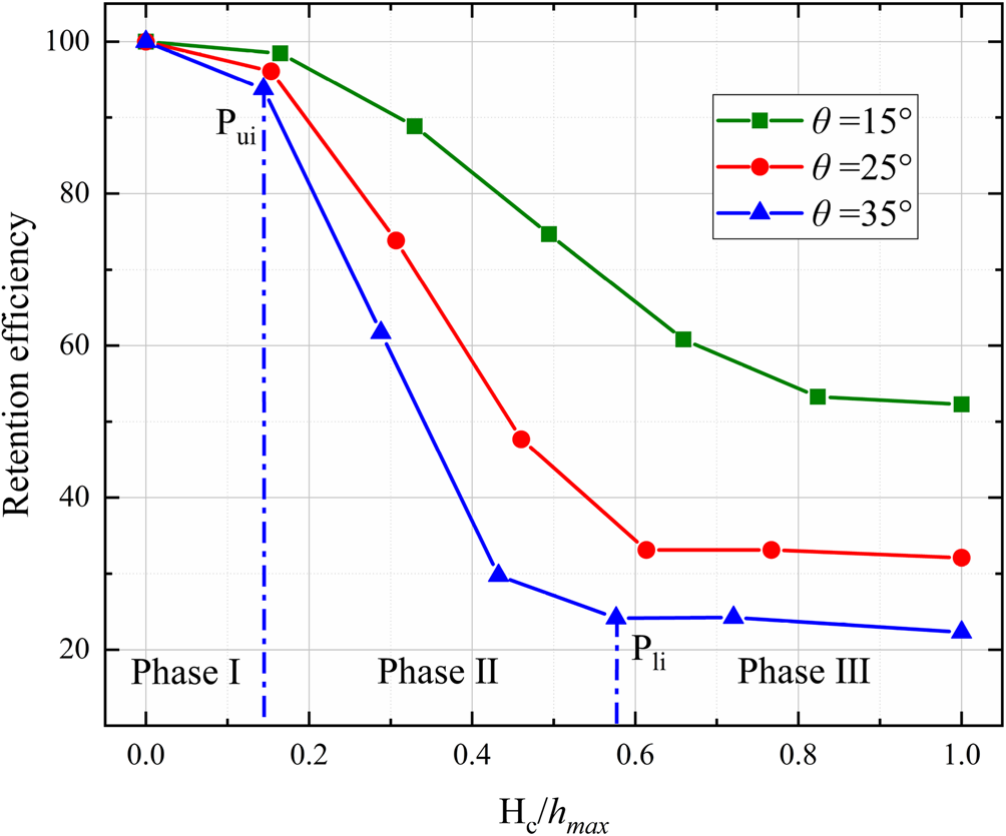
Dependence of *RE* on normalized bottom outlet height (${H}_{c}/{h}_{max}$) for various slope angles.

Understanding the dependence of *RE* on ${H}_{c}/{h}_{max}$ and the slope angle will help the designers choose the suitable size of bottom outlet to satisfy the trapping objective. As shown in Fig. 16, the relationship between *RE* and the normalized bottom outlet height exhibits a similar inverse S-shape trend for different slope angles. When *θ* = 35°, for example, *RE* decreases slowly before the upper inflection point P_ui_. Once ${H}_{c}/{h}_{max}$ is greater than P_ui_, *RE* decreases linearly with ${H}_{c}/{h}_{max}$ until the normalized bottom outlet height reaches the lower inflection point P_li_. The retention volume of the check dam is close to that of the free flow case when ${H}_{c}/{h}_{max}$ is greater than P_li_. Figure 16 shows that the slope angle has little influence on the upper inflection point P_ui_. A steeper slope tends to decrease the lower inflection point P_li_.

The variations in Q_r_ and *RE* imply that the regulation function of the open check dam performs well when $\frac{{H}_{c}}{{h}_{max}}<0.6$. During this phase, most of the debris mass will be blocked and deposited by the check dam. The bottom outlet of the check dam can then be regarded as a silo that allows the deposited debris mass to flow out of the check dam steadily under gravity. If $\frac{{H}_{c}}{{h}_{max}}>0.6$, only the upper part of the debris flow is blocked by the check dam, and the lower part of the debris flow can still pass through the bottom freely, which leads to a relatively low *RE*. This pattern implies that the regulation function of the open check dam may fail when $\frac{{H}_{c}}{{h}_{max}}>0.6$.

### Effect of outlet height on energy breaking efficiency

**Figure 17:**
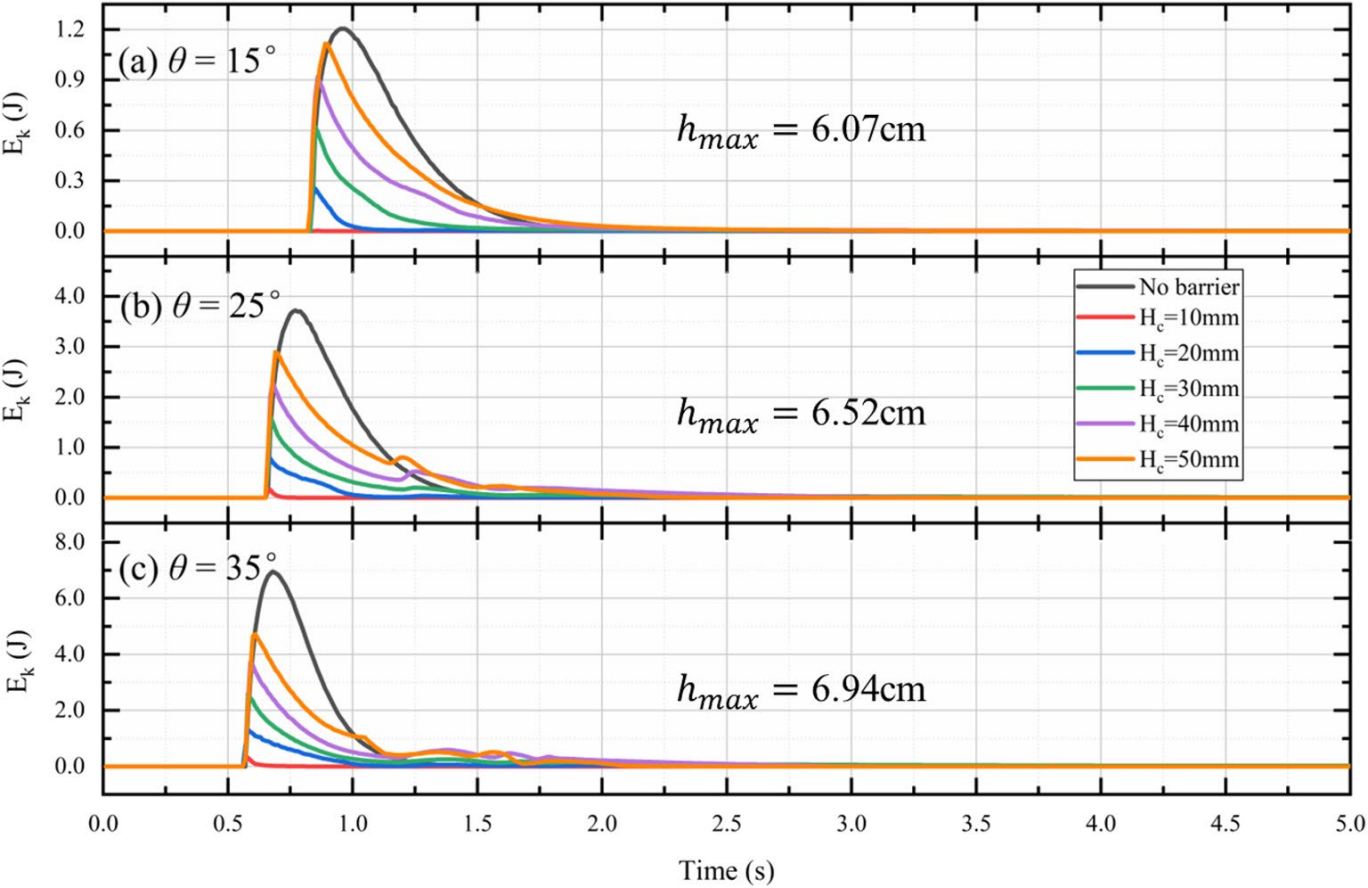
Evolution of outflow kinetic energy at different slope angles with different bottom outlet sizes.

Another important function of a check dam with a bottom outlet is to dissipate the kinetic energy carried by the debris flow. To study the effect of the bottom outlet on the energy dissipation induced by the check dam, the evolution of outflow kinetic energy is now investigated. The evolution of kinetic energy in the outflow at different slope angles with different bottom outlet sizes is plotted in Fig. 17. For test I15, for example, the outflow kinetic energy quickly reaches the maximum at t = 0.97 s and then gradually decreases. This implies that the flow front carries more kinetic energy than the tail. A steeper slope implies that more kinetic energy is carried by the flow front, as shown in Fig. 17.

Figure 17 shows that the maximum ${E}_{k}$ for cases with a check dam is smaller than for the free flow cases. This indicates that the check dam effectively decreases the kinetic energy of the flow front. The energy breaking efficiency (*EB*) is used to evaluate the energy dissipation capacity of the check dam. This can be defined as ^37^:

29 $$\begin{array}{*{20}c} {EB = \frac{{\int E_{k}^{freeflow} dt - \int E_{k}^{check dam} dt}}{{\int E_{k}^{freeflow} dt}},} \\ \end{array}$$

where ${E}_{k}^{freeflow}$ is the outflow kinetic energy of the free flow case and ${E}_{k}^{check dam}$ is the outflow kinetic energy when a check dam is installed.

**Figure 18:**
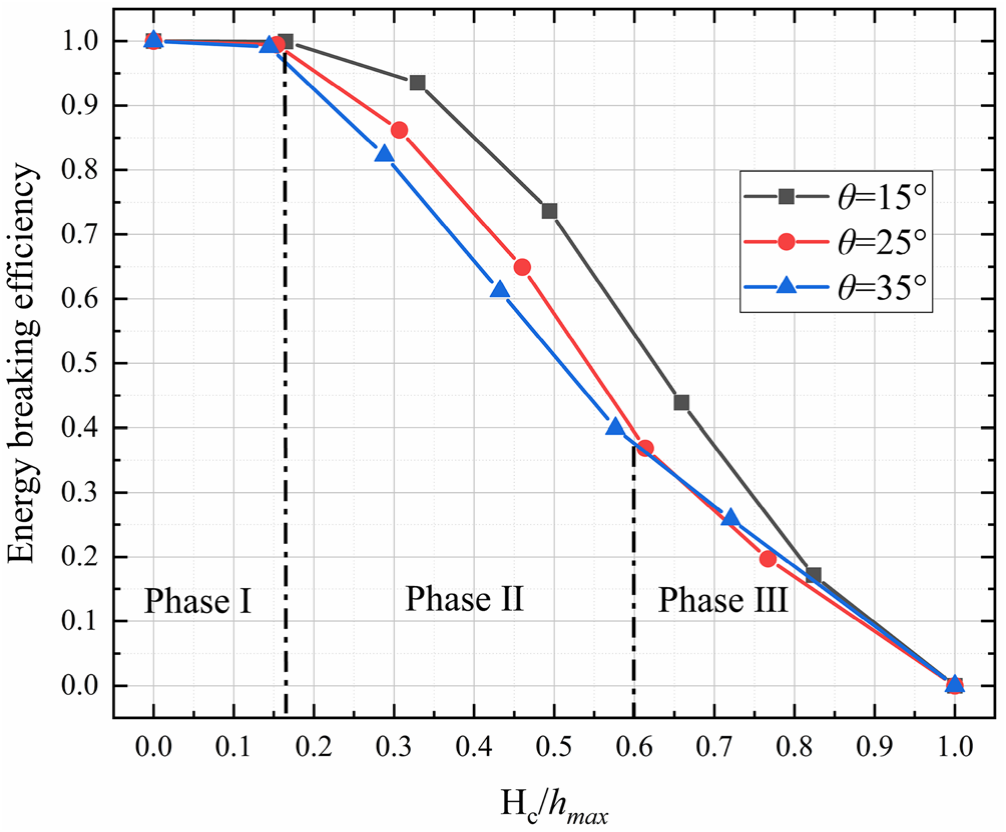
Relationship between *EB* and normalized bottom outlet height (${H}_{c}/{h}_{max}$) for various slope angles.

The relationship between *EB* and the normalized bottom outlet height (${H}_{c}/{h}_{max}$) for various slope angles is plotted in Fig. 18. Nearly all of the kinetic energy carried by the debris flow is blocked by the check dam until $\frac{{H}_{c}}{{h}_{max}}<0.15$, as shown in Phase I of Fig. 18. The drag force provided by the check dam bottom and flume base rapidly decelerates the debris flow as it passes through the bottom outlet. The flow inertia has little influence on the outflow after impact has occurred. This results in a very small outflow rate, as shown in Figs. 13, 14. The debris mass is then quickly deposited upstream of the check dam, leading to a very high *RE* (as shown in Fig. 16) and *EB* (as shown in Fig. 18).

*EB* decreases as ${H}_{c}/{h}_{max}$ increases. For $0.15<\frac{{H}_{c}}{{h}_{max}}<0.6$, a steeper slope results in lower *EB* at a given value of ${H}_{c}/{h}_{max}$, as shown in Phase II of Fig. 18. During this phase, the outflow is governed by flow inertia and the drag force provided by the check dam bottom and flume base. Thus, the outflow rate, *RE*, and *EB* are all affected by the slope angle and normalized bottom outlet height.

For $\frac{{H}_{c}}{{h}_{max}}>0.6$, *EB* is very low. The slope angle has a negligible influence on *EB* compared with the normalized bottom outlet height. In this case, only the upper part of the debris flow is blocked by the check dam, while the lower part can pass freely through the bottom outlet. The debris flow is governed by the inertia force in this instance, and the kinetic energy of the debris flow is largely dissipated by the block mechanism induced by the check dam. Thus, *EB* is mainly governed by ${H}_{c}/{h}_{max}$.

### Discussion

The influence of height of the bottom outlet on the dry granular flow has been studied by Choi et al.^7^ and Shen et al.^8^. In their studies, the ratio between the height of the bottom outlet ${H}_{c}$ and particle diameter *D* was chosen to be indicator to evaluate the influence of the check dam on the mobility of the debris flow. However, the results of current work show that the normalized height of the bottom outlet (${H}_{c}/{h}_{max}$) can be used as an indicator to evaluate the influence of the check dam on the mobility of the debris flow.

When dry granular flow impacts on the check dam with bottom outlets, the relationship between height of the bottom outlet and particle diameter plays an important role in the occurrence of jamming of the bottom outlets. However, the interaction between viscous debris flows and check dam with bottom outlets is governed by the hydraulic control mechanism. The height of bottom outlets controls the ratio between upward jet and downstream jet. Thus, the normalized height of the bottom outlet (${H}_{c}/{h}_{max}$) has a great influence on the mobility of viscous debris flow.

For low normalized bottom outlet heights ($\frac{{H}_{c}}{{h}_{max}}<0.15$), the check dam may produce a relatively high jump height, *RE*, and *EB*. At this stage, the performance of the check dam is similar to that of a closed check dam. The retention volume of the check dam becomes saturated after several debris flow events.

For high normalized bottom outlet heights ($\frac{{H}_{c}}{{h}_{max}}>0.6$), *RE* and the residual outflow rate of the check dam are very close to those in the case of free flow. This indicates that the discharge regulation and sediment trapping functions of the check dam may fail in the case of high normalized bottom outlet heights.

For median normalized bottom outlet heights ($0.15<\frac{{H}_{c}}{{h}_{max}}<0.6$), the jump height is significantly reduced by the bottom outlet compared with the case of a closed check dam. The kinetic energy and peak outflow of the debris flow are significantly reduced by the bottom outlet compared with the free flow case. Moreover, the check dam can temporarily intercept and retain part of the sediment, which eventually flows downstream through the bottom outlet. In general, the numerical tests show that when the normalized bottom outlet height is in the median range considered in this study, the energy breaking, flow regulation, and sediment trapping functions of the check dam operate effectively.

Although the numerical results provide useful design suggestions, this study still has some limitations. For instance, the debris flow was modeled as a homogeneous non-Newtonian fluid. Natural debris flows are always non-homogeneous because of the vertical profile of the particle distribution in the debris flow formation region^38^. The influence of the spatial variability of the fluid properties was not considered in this study. In addition, the interaction between large pieces of wood, boulders, and a check dam with a bottom outlet was not covered in this study. Given these limitations, further efforts are needed to validate the findings of this study in terms of the natural scale and material of debris flows. However, this study provides a basis for the rational design of check dams with bottom outlets.

## Conclusions

In this study, the interaction between debris flows and check dams with bottom outlets has been studied via flume tests using the 2D SPH method. The effects of the bottom outlet on the jump height, discharge, sediment trapping, and energy breaking were investigated. The findings from this study are as follows:

1. The jump height is influenced by the normalized height of the bottom outlet and the Froude number of the viscous debris flow. Based on the numerical results, the jump height decays with increasing normalized height of the bottom outlet and this trend can be approximated by a power low function.
2. For viscous debris flows, there is a strong positive correlation between the normalized height of the bottom outlet and the normalized peak outflow rate. When the normalized bottom outlet height is less than 0.5, the residual outflow rate increases with increasing slope angle and normalized bottom outlet height. When the normalized bottom outlet height is greater than 0.5, the residual outflow rate decreases with increasing normalized bottom outlet height.
3. For a given slope angle, *RE* and the normalized bottom outlet height exhibit an inverse S-shaped trend. The check dam can retain more than 90% of the debris flow if the normalized bottom outlet height is less than 0.15. If the normalized height of the bottom outlet exceeds 0.6, the discharge regulation and sediment trapping functions of the check dam may not operate effectively.
4. *EB* decreases with increases in the slope angle and the normalized bottom outlet height.

In summary, a median normalized bottom outlet height is recommended so that the energy breaking, flow regulation, and sediment trapping functions of check dams with bottom outlets operate effectively. This work provides a basis for the rational design of check dams with bottom outlets. Further efforts are needed to overcome the limitations of this study to further improve the design of check dams with bottom outlets.

## Supplementary Information

Supplementary Information.

## Data Availability

The data that support the findings of this study are available from the corresponding author upon request.
