# Supplementary material for: Numerical investigation on the role of check dams with bottom outlets in debris flow mobility by 2D SPH
Source: Sci Rep. 2022 Nov 28;12:20456. doi: 10.1038/s41598-022-24962-4 (PMC9705551; doi:10.1038/s41598-022-24962-4)
Supplement: Supplementary file 1 — Supplementary Information. [file 41598_2022_24962_MOESM1_ESM.docx]

**Title: Numerical investigation on the role of check dams with bottom outlets in debris flow mobility by 2D SPH**

Hao Shi^a^, Yu Huang^a,b*^_,_ Dianlei Feng^c^

1. Department of Geotechnical Engineering, College of Civil Engineering, Tongji University, Shanghai 200092, China
2. Key Laboratory of Geotechnical and Underground Engineering of the Ministry of Education, Tongji University, Shanghai 200092, China
3. Department of Hydraulic Engineering, College of Civil Engineering, Tongji University, Shanghai 200092, China

^*^Corresponding Author: Yu Huang (yhuang@tongji.edu.cn)

**Supplemental Content Legend**

**Figure S1. Snapshots of test I25 at typical instants: (a) t=0.00 s; (b) t=0.62 s; (c) t=0.80 s; (d) t=1.20 s; (e) t=5.00 s.** ($h_{max}$=6.52cm, $\bar{u}_{max}$=2.22, $Fr$=2.92)

**Figure S1**
